# Isolation of *Tibet orbivirus* from *Culicoides* and associated infections in livestock in Yunnan, China

**DOI:** 10.1186/s12985-017-0774-9

**Published:** 2017-06-08

**Authors:** Jinglin Wang, Huachun Li, Yuwen He, Yang Zhou, Aiguo Xin, Defang Liao, Jinxin Meng

**Affiliations:** Yunnan Animal Science and Veterinary Institute, Qinglongshan Jindian PanLong District Kunming, Kunming, Yunnan province 650224 People’s Republic of China

**Keywords:** *Tibet orbivirus*, Isolation, *Culicoides*, Whole genome analysis, Infection in animals

## Abstract

**Background:**

*Culicoides*-borne orbiviruses, such as *bluetongue virus* (BTV) and *African horse sickness virus* (AHSV), are important pathogens that cause animal epidemic diseases leading to significant loss of domestic animals. This study was conducted to identify *Culicoides*-borne arboviruses and to investigate the associated infections in local livestock in Yunnan, China.

**Methods:**

*Culicoides* were collected overnight in Mangshi City using light traps during August 2013. A virus was isolated from the collected *Culicoides* and grown using baby hamster kidney (BHK-21), Vero, Madin-Darby bovine kidney (MDBK) and *Aedes albopictus* (C6/36) cells. Preliminary identification of the virus was performed by polyacrylamide gel (PAGE) analysis. A full-length cDNA copy of the genome was amplified and sequenced. Serological investigations were conducted in local cattle, buffalo and goat using plaque-reduction neutralization tests.

**Results:**

We isolated a viral strain (DH13C120) that caused cytopathogenic effects in BHK-21, Vero, MDBK and C6/36 cells. Suckling mice inoculated intracerebrally with DH13C120 showed signs of fatal neurovirulence. PAGE analysis indicated a genome consisting of 10 segments of double-stranded RNA that demonstrated a 3–3–3–1 pattern, similar to the migrating bands of *Tibet orbivirus* (TIBOV). Phylogenetic analysis of the viral RNA-dependent RNA polymerase (Pol), sub-core-shell (T2, and outer core (T13) proteins revealed that DH13C120 clustered with TIBOV, and the amino acid sequences of DH13C120 virus shared more than 98% identity with TIBOV XZ0906. However, outer capsid protein VP2 and outer capsid protein VP5 shared only 43.1 and 79.3% identity, respectively, indicating that the DH13C120 virus belongs to TIBOV, and it may represent different serotypes with XZ0906. A serosurvey revealed the presence of neutralizing antibodies with 90% plaque-reduction neutralization against TIBOV DH13C120 in local cattle (44%), buffalo (20%), and goat (4%). Four-fold or higher levels of TIBOV-2-neutralizing antibody titers were detected between the convalescent and acute phases of infection in local livestock.

**Conclusions:**

A new strain of TIBOV was isolated from *Culicoides*. This study provides the first evidence of TIBOV infection in livestock in Yunnan, China, and suggests that TIBOV could be a potential pathogen in livestock.

## Background

The viral genus *Orbivirus* within the family *Reoviridae* has a genome consisting of 10 segments of double-stranded (ds) RNA. It contains 22 recognized virus species and 10 unclassified viruses [1]. BTV and AHSV negatively impact animal husbandry and veterinary health. In addition, *Equine encephalosis virus* (EEV), *Epizootic hemorrhagic disease virus* (EHDV), *Palyam virus* (PALV) and *Peruvian horse sickness virus* (PHSV) are important pathogens causing epidemic diseases in animals. In these orbiviruses, although PHSV is mosquito-borne, most of these viruses are carried by *Culicoides* [2, 3]. Therefore, *Culicoides* is considered a potent vector of important animal arbovirus diseases, which cause major economic losses in domestic animals [2, 4–6].

The TIBOV was first isolated from *Anopheles maculatus* mosquitoes collected in 2009 from Motuo County, Tibet, China [7]. Subsequently, TIBOV was isolated from *Culex fatigan* mosquitoes and *Culicoides* specimens collected from Guangdong and Yunnan, respectively [8]. Here, we report a new TIBOV named DH13C120, isolated from *Culicoides*. Sequence analysis of its entire genome and evidence of DH13C120 infection in local livestock were obtained from samples collected in the southwest border area of Yunnan Province in 2013.

## Methods

### Collection of *Culicoides* specimens

Specimens were collected during August 2013 from the suburb of Manbing Town in Mangshi City, Dehong Prefecture, Yunnan Province. Samples were collected overnight using light traps (12 V, 300 mA; Wuhan Lucky Star Environmental Protection Tech. Co., Ltd., Hubei, China). Captured *Culicoides*, killed by freezing at −20 °C for 20 min and identified by using morphologic characteristics, were pooled into groups of approximately 100 insects and stored in liquid nitrogen until viral isolation.

### Isolation of viruses

*Culicoides* pools, which included midges that were engorged or not engorged, were removed from liquid nitrogen and immediately homogenized and centrifuged as reported previously [9]. The supernatants were used to inoculate monolayers of baby hamster kidney (BHK-21) cells, Vero cells (from the China National Institute for Viral Disease Control and Prevention, Chinese Center for Disease Control and Prevention, Beijing, China), and Madin-Darby bovine kidney (MDBK) cells (From Yunnan biological pharmaceutical factory, Kunming, China) at 37 °C; *Aedes albopictus* (C6/36) cells (from the National Institute for Viral Disease Control and Prevention) were inoculated at 28 °C. The cells were observed daily (days 1–7 post-inoculation) for cytopathic effects (CPEs).

### Assay of neurovirulence in suckling mice

Aliquots of 30 μL cell culture material (100 plaque-forming units, PFU/mL) were inoculated intracerebrally into 1-day-old suckling mice, and 30 μL of Dulbecco’s modified Eagle’s medium (DMEM) was similarly applied in the control group, with eight suckling mice in each group. Symptoms (or the time of death) in the virus-treated neonatal mice were recorded each day for 14 days post-inoculation.

### Polyacrylamide gel electrophoresis (PAGE)

To reveal the number of dsRNA segment and the genome pattern, viral RNA was extracted using RNAiso Plus (TaKaRa, Dalian, China) according to the manufacturer’s protocols, and then separated on RNA-PAGE as described previously [9–11].

### Full genome sequencing

The virus genome was amplified by full-length amplification of cDNA (FLAC), as described previously [9, 12, 13]. Briefly, viruses were propagated in BHK-21 cells, and total RNA was extracted using RNAiso Plus (TaKaRa) according to the manufacturer’s protocols. Single stranded (ss) RNAs were removed by precipitation with 2 M LiCl (Sigma-Aldrich, St Louis, MO, USA), and dsRNAs were precipitated by the addition of 2.5 volumes of isopropanol and 1 volume of 7.5 M ammonium acetate. The dsRNAs were subjected to 1% agarose gel electrophoresis (AGE) (7 V/cm, for 1 h) in TAE buffer (40 mMTris-acetate, 1 mM EDTA, pH 8.0) and purified from the agarose gel using MinElute gel extraction kits (Qiagen, Hilden, Germany).

An anchor primer, PC3-T7 loop (synthesized by Sangon, Shanghai, China), similar to that described by Maan et al. [13], was ligated to the viral dsRNA. Ligation reactions were carried out in a total volume of 30 μL, as described previously [9, 12]. The ligated dsRNA was purified using MicroElute RNA Cleanup kits (Omega Bio-Tek Inc., Norcross, GA, USA), denatured with dimethyl sulfoxide (DMSO) for a final concentration of 15% (v/v), heated in boiling water for 2 min, and snap-frozen in an ice-water slurry. The full-length complementary DNAs (cDNAs) of 10 viral dsRNA segments were synthesized in a total volume of 50 μL using a PrimeScript II high-fidelity reverse transcription polymerase chain reaction (RT–PCR) kit (TaKaRa) according to the manufacturer’s recommendations. Amplification of the cDNA was carried out with primer PC2 in a total volume of 50 μL, as described previously [9, 12]. The PCR products were viewed after separation on 1% agarose gels in 1× TAE buffer containing a nucleic acid dye, and purified using TaKaRa DNA fragment purification kits (ver. 2.0; TaKaRa). The gel-purified fragments were cloned into the pMD19-T vector (TaKaRa), and introduced into chemically competent *Escherichia coli* DHα5 cells (TaKaRa). Single colonies were cultured and sequenced by the Shenzhen Huada Genome Institute, Shanghai, China. We performed 2 sequencings from the same RNA extraction and these sequences (that include the differences) were cover 2.5 times.

### Sequence analysis and phylogenetic comparisons

Initial sequence assembly and analyses were conducted using the DNAStar software package (ver. 4.0; DNASTAR Inc., Madison, WI, USA). Homology and alignment analysis was performed using Clustal X (ver. 2.1) [9] (http://www.directoryofshareware.com/preview/clustalx/) and MegAlign (DNASTAR Inc.). Phylogenetic and evolutionary analyses were conducted using MEGA 5.1 (http://www.megasoftware.net/) based on the neighbor-joining technique and 1,000 bootstrap replications [9].

### Serological investigations of antibodies against TIBOV isolate DH13C120

#### Sample collection

Buffalo serum samples were collected in Mangshi City, Dehong Prefecture, Yunnan Province in 2009. The separated serum samples were stored at −20 °C until tested. In 2013, three collection sites were chosen, in Mangshi City, Jiangcheng County of Pu’er City, and Shizong County of Qiujing City. Sentinel animals were picked from and existing population if they were first shown to serologically naïve on the May. Ten cattle and five goats were chosen as sentinel animals for each collection site, and serum samples were collected monthly and stored at −20 °C until tested.

### Plaque-reduction neutralization tests

Serum samples were tested for neutralizing antibodies against TIBOV isolate DH13C120 by 90% plaque-reduction neutralization tests (PRNT 90) using standard methods. Samples were tested with serial two-fold dilutions from 1:10 to 1:1,280. Diluted samples were mixed with equal volumes of culture medium 1× MEM (pH = 7.4) containing DH13C120 (100 PFU) and incubated at 37 °C in a 5% CO_2_ for 1 h. Six-well plates of confluent BHK-21 cells were inoculated with the serum–virus mixtures and incubated at 37 °C in a 5% CO _2_ incubator for 1 h. Plates were overlaid with 3 mL of the same medium containing 0.8% agarose, and again with 3 mL of a second overlay medium containing neutral red vital stain (Sigma-Aldrich). The neutralizing antibody titer was identified as the highest serum dilution that reduced the number of virus plaques in the test by ≥ 90%. The samples were considered to be positive when titers were ≥ 20. A ratio of TIBOV PRNT 90 titer between two samples of a same animal ≥ 4 was considered to confirm the presence of a TIBOV infection [14].

## Results

### Isolation of viruses

*Culicoides* were processed to isolate viruses in BHK-21, Vero and C6/36 cells. A CPE was observed with one pool (DH13C120) collected from Dehong. Isolate DH13C120 caused CPEs in BHK-21, Vero, MDBK and C6/36 cells at 48, 48, 144, and 120 h post-infection, respectively. The characteristic CPE in BHK-21, Vero and C6/36 cells infected with DH13C120 was rounding and desquamation, whereas that in MDBK cells showed vacuolization and desquamation.

### Assay of neurovirulence in suckling mice

Suckling mice inoculated intracerebrally with DH13C120 showed signs of tremor and stiff necks at 48 h, and died within 72 h.

### Gel separation

**Fig. 1:**
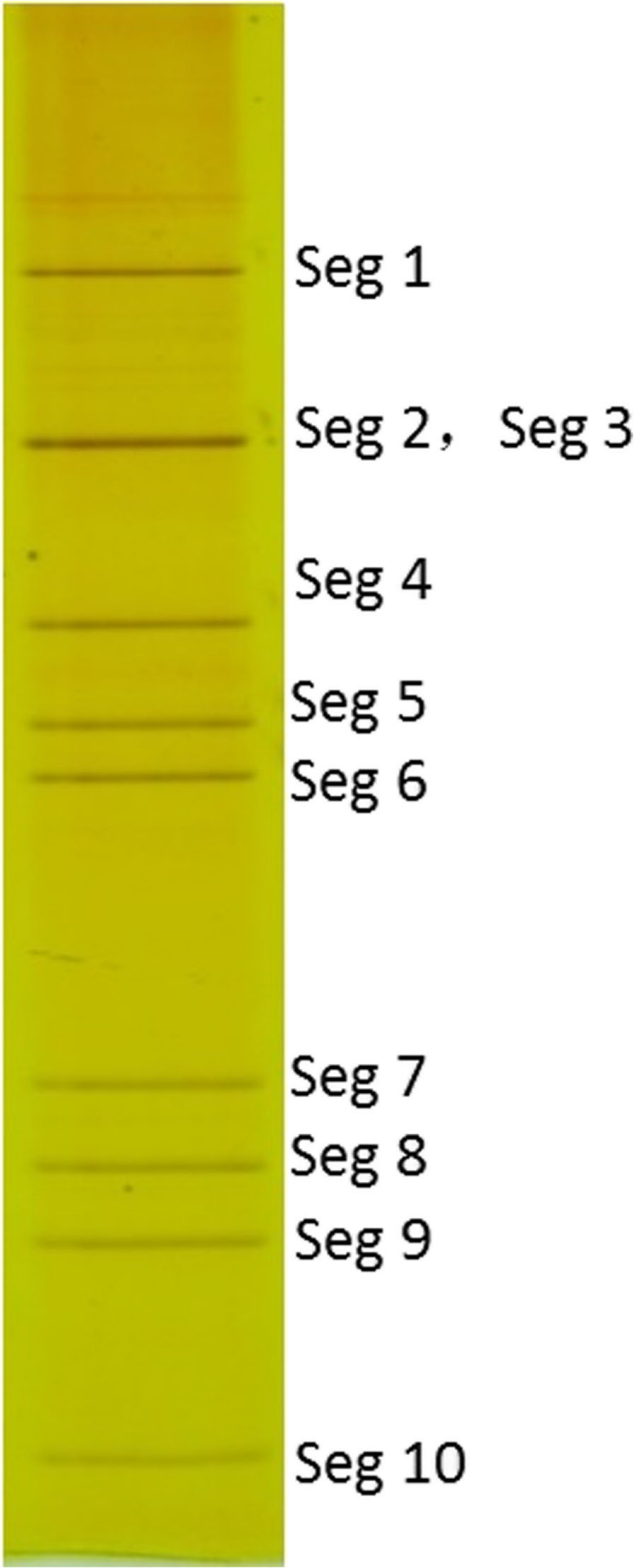
Electrophoretic patterns of the dsRNA of virus DH13C120 by polyacrylamide gel electrophoresis

Separation by PAGE revealed that DH13C120 is a 10-segment dsRNA virus, with a segment pattern of 3–3–3–1 (Fig. 1).

### Genome organization and characteristics of DH13C120 virus

A full length cDNA copy of the DH13C120 virus genome was obtained by FLAC and sequencing, with the length of segments 1–10 ranging between 3,740 nucleotides (nt) (Seg-1) to 788 nt (Seg-10) (Table 1). The sequences of the 10 segments were deposited in the NCBI GenBank with the accession numbers KU754026-KU754035, as indicated in Table 1. Compared with the TIBOV XZ0906 strain, the DH13C120 virus has 12 nt insertions in the open reading frame and 1 nt insertion in the 3′ untranslated region (UTR) in Seg-2, and 5 nt insertions in the 5′ UTR and 1 nt insertion in the 3′ UTR in Seg-6. Conserved nt sequences of 6 nt were observed at the ends of the 5′ and 3′ UTRs (5′-GTAAAA^A^/_T_ and ^C^/_A_ACTTAC-3′), and 4 nt in the terminal sequences were reverse complementary. The 5′ and 3′ UTRs of DH13C120 comprised 3.80% of the total genome; the G + C content of DH13C120 was 42.59%, similar to *Culicoides*-borne *Orbivirus* species; and an Arg–Gly–Asp (RGD) motif [15–17] was observed at position 168–170 in the viral outer core protein T13 (VP7) of DH13C120.

**Table 1:** Lengths of dsRNA segments 1–10, encoded putative proteins, 5′and 3′NCRs of DH13C120 virus genome

Segment	Length (bp)	GC%	Protein (aa)	5′NCR (bp)	Terminal sequence (5′–3′)	3′NCR (bp)	Putative function	GenBank Accession no.
S1	3950	41.32	1304	11	GUAAAAUCA---AUACACUUAC	24	VP1: RNA-dependent RNA polymerase	KU754026
S2	2901	40.09	950	13	GUAAAAAUC---UUAAACUUAC	35	VP2: outer capsid protein; controls virus serotype; most variable protein; contains neutralizing epitopes	KU754027
S3	2769	44.42	899	17	GUAAAAUUU---AUACACUUAC	52	VP3(T2): forms sub-core capsid layer	KU754028
S4	1978	42.82	643	8	GUAAAAACA---UUACACUUAC	38	VP4: Cap; protein within the sub-core; shows capping activities and methyltransferase type 1 and type 2 activities	KU754029
S5	1775	43.83	554	31	GUAAAAAAG---UUACACUUAC	79	NS1: TuP; forms tubules of unknown function within the cytoplasm of infected cells	KU754030
S6	1642	43.42	526	31	GUAAAAAGA---UUUCACUUAC	30	VP5: outer capsid protein	KU754031
S7	1165	44.72	349	17	GUAAAAAUU---UUACACUUAC	98	VP7(T13): immunodominant major serogroup specific antigen	KU754032
S8	1142	41.94	359	20	GUAAAAAAU---UUAAACUUAC	42	NS2: single-stranded RNA-binding protein	KU754033
S9	1100	44.73	346	14	GUAAAAAAU---UAAAACUUAC	45	VP6: helicase for unwinding and binding to ssRNA and dsRNA; NTPase	KU754034
S10	832	41.47	234	21	GUAAAAAAG---CCCAACUUAC	106	NS3: involved in cell exit; glycoprotein	KU754035
		42.59	Consensus		GUAAAA^A^/_U_---------^C^/_A_ACUUAC			

### Phylogenetics

#### Phylogenetic analysis of DH13C120 virus compared with TIBOV (XZ0906, Fengkai and YN12246)

The sub-core-shell (T2) (VP3) protein of DH13C120 is encoded by segment 3. Phylogenetic analvysis showed that DH13C120 has a close relationship with TIBOV XZ0906, YN12246 (only partial nt sequence of the T2 protein) and Fengkai virus. Amino acid (aa) sequence analysis of the T2 protein revealed 96.3–99.9% sequence similarity between DH13C120 and the TIBOV species (XZ0906, YN1226 and *Fengkai virus*), which was above the threshold of 91% used for species determination by Attoui et al. [18]. This similarity confirmed that DH13C120 is a strain of TIBOV.

Sequence analysis of the outer capsid VP2 (encoded by segment 2) protein of TIBOV isolated from China (DH13C120, XZ0906 and the *Fengkai virus*) revealed that the DH13C120 virus isolated from *Culicoides* in Yunnan and the *Fengkai virus* isolated from mosquitoes in Guangdong (partial sequence) had sequence similarities of 96.8%/97.1% nt/aa, whereas XZ0906 isolated from mosquitoes in Tibet displayed similarities of 53.9%/43.1% nt/aa (Table 2).

**Table 2:** Comparison of each segment between virus DH13C120 and other Orbiviruses in nucleotide and amino acid identities

Segment	TIBOV (XZ0906)		TIBOV (YN1226)		TIBOV (Fengkai)		Pata virus		BTV-1		EHDV-2		CHUNV		YOUV	
	nt(%)	aa(%)	nt(%)	aa(%)	nt(%)	aa(%)	nt(%)	aa(%)	nt(%)	aa(%)	nt(%)	aa(%)	nt(%)	aa(%)	nt(%)	aa(%)
S1(VP1)	92.0	98.6	90.9^a^	98.5^a^	91	98.5	67.8	71.8	66.5	71.0	66.9	70.6	58.8	58	52.4	47
S2(VP2)	53.9	43.1	–	–	96.8^b^	97.1	46.2	26.5	44.0	25.2	45.7	23.3	44.1	21.9	42.4	21.8
S3(VP3)	96.5	99.9	79.5^a^	96.3^a^	96	99.8	69.4	77.8	68.4	75.2	69.4	76.0	60.7	57.6	47.4	38.0
S4(VP4)	96.6	98.0	–	–	96.1	98.3	61.0	63.0	62.5	64.6	62.0	64.3	53.6	41.0	48.8	48.2
S5(NS1)	96.8	99.8	–	–	93.4	99.7	52.7	45.2	49.9	39.1	49.9	40.7	41.6	27.6	36.4	25.9
S6(VP5)	70.4	79.3	–	–	94.8	98.9	61.1	62.9	59.4	59.4	60.0	61.3	51.2	45.5	45.1	30.9
S7(VP7)	98.9	99.7	–	–	93.5	99.1	66.0	68.2	61.6	60.2	61.9	61.3	51.6	40.6	36.4	23.6
S8(NS2)	88.4	97.2	–	–	96.8	98.3	60.9	41.5	59.5	31.3	59.8	35.6	53.9	32.0	46.7	26.0
S9(VP6)	96.4	94.2	–	–	96.4	94.2	61.6	46.3	44.5	33.9	59.5	38.9	41.2	30.4	38.1	31.2
S10(NS3)	98.4	99.6	–	–	85.5	94.5	60.7	55.4	57.6	54.8	59.2	53.5	36.7	35.0	38.0	25.8

Genbank accession numbers for virus sequences used for comparison: 1) TIBOV(XZ0906): KF746187- KF746196; 2) TIBOV (YN1226): KP099640(VP1), KP099641(VP3); 3) TIBOV (Fengkai): NC_027803- NC_027815; 4) Pata virus: JQ070386- JQ070395; 5) BTV-1: KF664123- KF664132; 6) EHDV-2: KM509050- KM509059; 7) CHUNV: NC_005986-NC_005995; 8) YOUV: NC_007656- NC_007665

^a^Part sequence;-:No corresponding sequence

^b^Fengkaivirus Seg-2: analyse only using 2779 nt of 5′-end sequence

#### Phylogenetic analysis of DH13C120 compared with other *Orbivirus* species

**Fig. 2:**
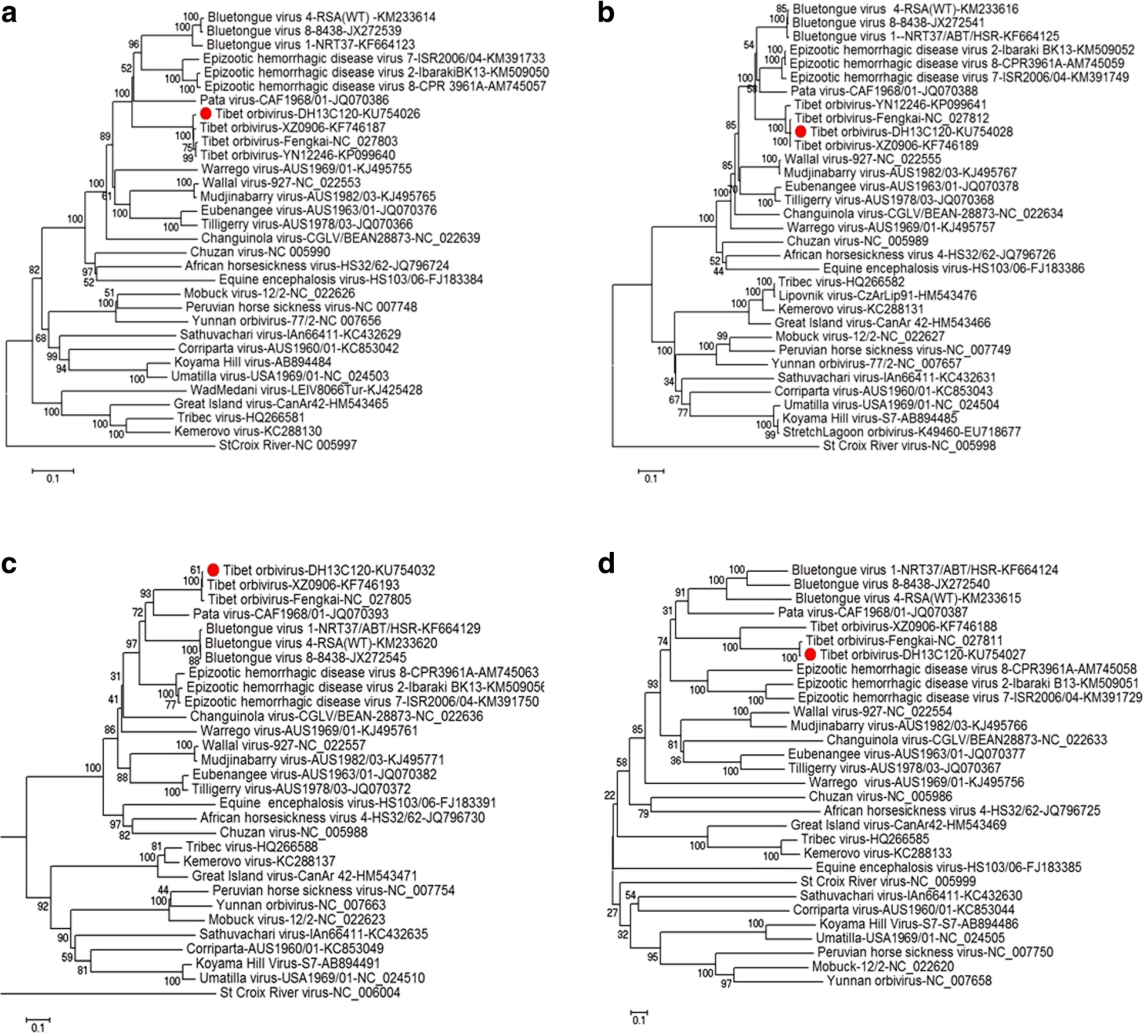
Phylogenetic analysis of the complete amino acid sequences of the corresponding viral proteins (VPs, see Table 1) of DH13C120 using representative members of the genus *Orbivirus*. **a** RNA-dependent RNA polymerase (Vol) (VP1); **b** T2 layer of core/subcore (VP3:Bluetongue virus (BTV), Epizootic hemorrhagic disease virus (EHDV), Tibet orbivirus (TIBOV), Pata virus, Wallal virus, Mudjinabary virus, Eubenangee virus, Warrego virus, Tilligerry virus, Changuinola virus, African horse sickness virus, Chuzan virus; VP2:Mobuck virus, Peruvian horse sickness virus, Yunnan orbivirus, Sathuvachai virus, Coriparta virus, Koyama Hill virus, Umatilla virus, Wadmedani virus, Great island virus, Tribec virus, Kemerovo virus, Stcroix River virus); **c** T13 sub-core capsid layer (VP7); **d** outer capsid protein (VP2)

To clarify the taxonomic position of DH13C120 with other *Orbivirus* species, we conducted phylogenetic analyses of the predicted aa sequences of RNA-dependent RNA polymerase (Pol) (VP1), T2 (VP3), and T13 (VP7) (Fig. 2). The phylogenetic trees revealed that the phylogeny was often correlated with a particular arthropod vector and could be classified into two groups: *Orbivirus* species transmitted by *Culicoides* (including BTV, AHSV, EHDV, *Wallal virus*, *Eubenangee virus*, *Warrego virus*, and PALV), and those transmitted by ticks (such as *Great island virus*, GIV) or mosquitoes (such as *Corriparta virus*, *Wongorr virus*, PHSV, and *Yunnan Orbivirus* (YUOV). Phylogenetic analysis showed that DH13C120 has a close relationship and similar coding pattern to other *Culicoides*-borne *Orbivirus* species. The Pol, T2, and T13 proteins of DH13C120 share 58–71.8%, 57.6–77.8%, and 40.6–68.2% aa sequence identity with the *Culicoides*-borne *Orbivirus* species, respectively, and 47%, 38%, and 23.6% aa identity with mosquito-borne YUOV, respectively.

### Prevalence of DH13C120 virus antibodies in local livestock

A total of 135 serum samples were collected from cattle, buffaloes and goats in Mangshi City, Jiangcheng and Shizong County of Yunnan. Neutralizing antibodies against DH13C120 virus were detected by PRNT 90 assays. Most of the positive samples were collected in Jiangcheng County, with the highest seroprevalence (90%) in cows. Additionally, positive sera were collected in Mangshi City (27%) and Shizong County (10%), but positivity rates in both areas were much lower than those in Jiangcheng County (Table 3).

**Table 3:** Results of PNPT90 antibody against TIBOV from cattle, buffalo and goat serum samples in the Yunnan

Species	SZ	DH	JC	Total
Cattle	1/10(10%)	12/30(40%)	9/10(90%)	22/50(44%)
Buffalo	NA	12/60(20%)	NA	12/60(20%)
Goat	0/5(0)	1/15(6.67%)	0/5(0)	1/25(4%)
Total	1/15(6.67%)	25/105(23.81%)	9/15(60%)	35/135(25.93%)

To understand the dynamics of infection by this orbivirus, five goats and ten cows were chosen as target animals at each of the three monitoring sites. Seroconversion of neutralization antibodies were detected in the target cattle (Table 4), with 9/10 in Jiangcheng County, 3/10 in Mangshi City, and only 1/10 in Shizong County. However, no seroconversion was detected in the target sheep. The period of seroconversion was from June to September (Table 4). This result illustrated that cattle are susceptible to the DH13C120 virus and may play an important role in viral diffusion and dissemination.

**Table 4:** Dynamic level of antibody against TIBOV DH13C120 strain in the target animal of 3 monitoring spots in Yunnan

NO.	Animal	Place	May	June	July	August	September	October	November	December
DH03	Cattle	Mangshi	<1:10	<1:10	<1:10	<1:10	1:20	1:80	1:80	1:160
DH05	Cattle	Mangshi	<1:10	<1:10	<1:10	<1:10	1:20	1:80	1:160	1:320
DH07	Cattle	Mangshi	<1:10	<1:10	<1:10	1:10	1:20	1:80	1:160	1:640
JC06	Cattle	Jiangcheng	<1:10	1:10	1:40	1:320	1:320	1:640	1:1280	
JC07	Cattle	Jiangcheng	<1:10	<1:10	1:10	1:40	1:80	1:80	1:80	
JC09	Cattle	Jiangcheng	<1:10	<1:10	<1:10	1:20	1:40	1:40	1:40	
JC10	Cattle	Jiangcheng	<1:10	<1:10	<1:10	<1:10	1:40	1:80	1:320	
JC11	Cattle	Jiangcheng	<1:10	<1:10	<1:10	1:20	1:320	1:640	1:640	
JC12	Cattle	Jiangcheng	<1:10	1:10	1:160	1:160	1:640	1:640	1:1280	
JC13	Cattle	Jiangcheng	<1:10	1:10	1:40	1:40	1:80	1:1280	1:640	
JC14	Cattle	Jiangcheng	<1:10	<1:10	<1:10	1:10	1:80	1:80	1:80	
JC15	Cattle	Jiangcheng	<1:10	<1:10	<1:10	1:20	1:40	1:640	1:640	
SZ03	Cattle	Shizong	<1:10	<1:10	<1:10	<1:10	1:10	1:20	1:20	

## Discussion

The parameters recognized by the International Committee on Taxonomy of Viruses for the polythetic definition of individual *Orbivirus* species include: the reassortment of genome segments; genome segment migration patterns shown by 1% agarose gel electrophoresis; conserved terminal nt sequences; serological cross-reactions; comparison of homologous genome segments or proteins by sequence analysis or cross-hybridization; host and vector ranges; and the nature of clinical signs induced by infection [19, 20]. In this study, some features of DH13C120 resembled the XZ0906 strain of TIBOV isolated from mosquitoes in Tibet [7] in terms of CPE, genome segment migration patterns, and conserved terminal nt sequences of the 5′ and 3′ UTRs, suggesting that DH13C120 belongs to the TIBOV species of the *Orbivirus* genus.

Conserved sequence from T2, Pol or T13 protein genes had previously been used for phylogenetic comparisons and taxonomic classification of *Orbivirus* [19, 21–23]. Among Orbivirus species, these proteins shared > 83%, > 73% and > 73% aa identity, respectively [18, 19, 22, 24]. DH13C120 and TIBOV shared 97% aa identity in the above-mentioned conserved proteins. In contrast, they showed lower levels of aa identity (<77.8%) with T2, Pol and T13 protein in other *Orbivirus* species. These data confirmed that DH13C120 is closely related to TIBOV. However, DH13C120 and TIBOV shared only 43.1% aa identity in the outer capsid protein VP2, and 79.3% aa identity in the outer capsid protein VP5. The outer capsid protein VP2 of the Orbivirus genus is an outer capsid protein; outer capsid protein VP2 is highly variable and determines viral serotype [21, 25, 26]. A pairwise alignment of outer capsid protein VP2s from different serotypes of BTV, EHDV and AHSV indicated variations in the ranges of 28.3–64%, 31.1–76.7%, and 46–52% aa, respectively [26–28]. Based on these data, although the DH13C120 virus and TIBOV belong to the same species, they may represent different serotypes.

In terms of vectors, the genus *Orbivirus* contains *Culicoides*-borne, mosquito-borne and tick-borne viruses [21]. Although TIBOV has been isolated from both *Culex* and *Culicoides*, phylogenetic analysis of T2, polymerase proteins and T13 proteins reveals that TIBOV is more closely related with the *Culicoides*-borne *Orbivirus*. Furthermore, Pol, T2, T13 proteins of DH13C120 share higher aa sequence identities with the *Culicoides*-borne *Orbivirus* than the mosquito- or tick-borne *Orbivirus* species, suggesting that *Culicoides* may be biological vectors of *DH13C120 virus*.

Previous studies have shown that the noncoding region (NCR) comprises 5.03–5.695% of *Orbivirus* genomes in the mosquito-borne group, 4.47–4.9% in the tick-borne group, and 3.5–4.1% in the *Culicoides*-borne group [22]. Analysis of the DH13C120 genome showed that the NCR comprises 3.80% of the genome, within the range of the *Culicoides*-borne group. In addition, the G + C content was 42.59% in DH13C120, which was within the range for *Culicoides*-borne *Orbivirus* species (from 39.89% in Chuzan virus to 45.89% in EEV) [19, 22], but more than that in the mosquito-borne species (from 36.72% in PHSV to 41.55% in YUOV) and less than that in the tick-borne species (from 57.29% in GIV to 51.93% in *St Croix River virus*). These data suggested that DH13C120 virus is more or most related to viruses vectored by *Culicoides spp*.

For *Culicoides*-borne *Orbivirus* species, such as BTV, EHDV, and AHSV, a highly conserved RGD motif was present at position 168–170 of VP7 (T13, core-surface protein), which could play a role in the attachment of core particles to *Culicoides* cells [15–17]. An RGD motif was observed at position 168–170 in T13(VP7) of TIBOV, DH13C120, XZ0906, and *Fengkai virus*, reflecting its closer overall similarity to BTV and EHDV, and potentially implicating *Culicoides* spp. as biological vectors.

In 1995, 17 strains of *Orbivirus* were first isolated from ticks, birds, rats, and livestock in the Yadong and Cuona areas of Tibet, with typical CPE in BHK-21 cells and lethal illness in suckling mice. Antibodies against the *Orbivirus* isolates were detected in local residents with a seroprevalence of 26%, suggesting that the new *Orbivirus* isolates could potentially be a novel animal pathogen in China [29, 30]. Recently, a new species, TIBOV, which could also generate CPE in BHK-21 cells, was isolated from mosquitoes and *Culicoides* in Yunnan, Guangdong Province and Tibet [7, 8]. In the present study, a new serotype of TIBOV was isolated from *Culicoides* in Yunnan, and high levels of neutralization antibodies were detected in cattle serum from three areas in Yunnan Province. Moreover, neutralization antibodies against DH13C120 were detected in target animals at all three monitoring sites, with the highest positivity rate being 92%. In addition, neutralization antibody titers that increased more than four-fold were detected in target cattle, and sheep inoculated with DH13C120 experienced fever (data not shown), suggesting that DH13C120 can infect livestock and is a potential pathogen leading to livestock disease.

In this study, DH13C120 was isolated from *Culicoides* collected in cattle corrals, and neutralization antibodies were detected in animals from the same areas. Seroconversion in animals occurred from June to September, which matched the seasonal peak of *Culicoides* numbers. These results suggest that *Culicoides* is the main vector of this new serotype of TIBOV, and cattle form the natural host reservoir. The cattle in these localities have high economic value, and most cattle are bred together in herds numbering over 100. The density of *Culicoides* was high in these cattle corrals, facilitating circulation of the new serotype. To understand the impact of this new TIBOV serotype, further studies are required to characterize its dissemination and pathogenicity.

## Conclusions

This investigation represents the isolation of TIBOV from *Culicoides*, and provides the first evidence of TIBOV DH13C120 strain infections in local livestock bred in the southwest border area of Yunnan, China. The results suggest that TIBOV isolated from *Culicoides* in yunnan, might be a pathogen causing livestock disease.

## Abbreviations

AHSV: *African horse sickness virus*
BHK-21: Baby hamster kidney
BTV: *bluetongue virus*
C6/36: *Aedes albopictus*
cDNAs: Complementary DNAs
CPEs: Cytopathic effects
DMEM: Dulbecco’s modified Eagle’s medium
DMSO: Dimethyl sulfoxide
EEV: *Equine encephalosis virus*
EHDV: *Epizootic hemorrhagic disease virus*
FLAC: Full-length amplification of cDNA
GIV: *Great island virus*
MDBK: Madin-Darby bovine kidney
PAGE: Polyacrylamide gel
PALV: *Palyam virus*
PFU: Plaque-forming units
PHSV: *Peruvian horse sickness virus*
PRNT 90: 90% plaque-reduction neutralization tests
RT–PCR: Reverse transcription polymerase chain reaction
TIBOV: *Tibet orbivirus*
UTR: Untranslated region
YUOV: *Yunnan Orbivirus*
